# Streptozotocin-induced Alzheimer's disease investigation by one-dimensional plasmonic grating chip

**DOI:** 10.1038/s41598-022-26607-y

**Published:** 2022-12-19

**Authors:** Hussam Jawad Kadhim, Haider Al-Mumen, H. H. Nahi, S. M. Hamidi

**Affiliations:** 1 Magneto‑plasmonic Lab, Laser and Plasma Research Institute, Shahid Beheshti University, Tehran, Iran; 2 Department of Electrical Engineering, College of Engineering, University of Babylon, Babylon, Iraq; 3 College of Veterinary Medicine, Al-Qasim Green University, Babylon, Iraq

**Keywords:** Neural ageing, Applied optics, Optical materials and structures, Optical techniques

## Abstract

Recently, there has been significant interest in researching brain insulin resistance as it has been hypothesized that it may play a role in the progression of Alzheimer's disease. Alzheimer’s disease (AD) is brain dementia that contributes to damage to the neuron cells and then patient death. This dementia is ranked as the fifth more dangerous disease in the world. Streptozotocin (STZ) is used to induce Alzheimer’s disease experimentally. STZ is toxic to the pancreatic beta cells and induces insulin resistance. Neuroplasmonin techniques have been used to investigate the ability of STZ on the activity of cultured neuron cells. Neuroplasmonic is a novel technology that combines nanotechnology and biosensor. This technique has been used to record neuron signals in vivo and in vitro. Also, it has many facilities such as label-free detection, real-time analysis, biological compatibility, small sample, high throughput, and low detection limit. In this paper, we introduce a one-dimensional electro-plasmonic nanograting platform that consists of a straight nanorod of gold embedded in a dielectric layer of polycarbonate. The chip is connected with an externally applied voltage to induce tunable PIT and increase the sensor sensitivity. To evaluate the sensing performance of the electro-plasmonic sensor, this chip was cultured with Human Nucleus Pulposus Cells (HNPC). The first step was to measure the neuron cell activity in a healthy case. The next step was to measure the activity of neuron cells injected with different concentrations of STZ (0.5, 1, 2 mM) to induce the formation of Alzheimer’s disease in the cultured neuron cells. The results indicated that the electro-plasmonics sensor had a high sensitivity to the cells' activity and showed good results for the effecting STZ on the neuron cell’s activities.

## Introduction

**Figure 1:**
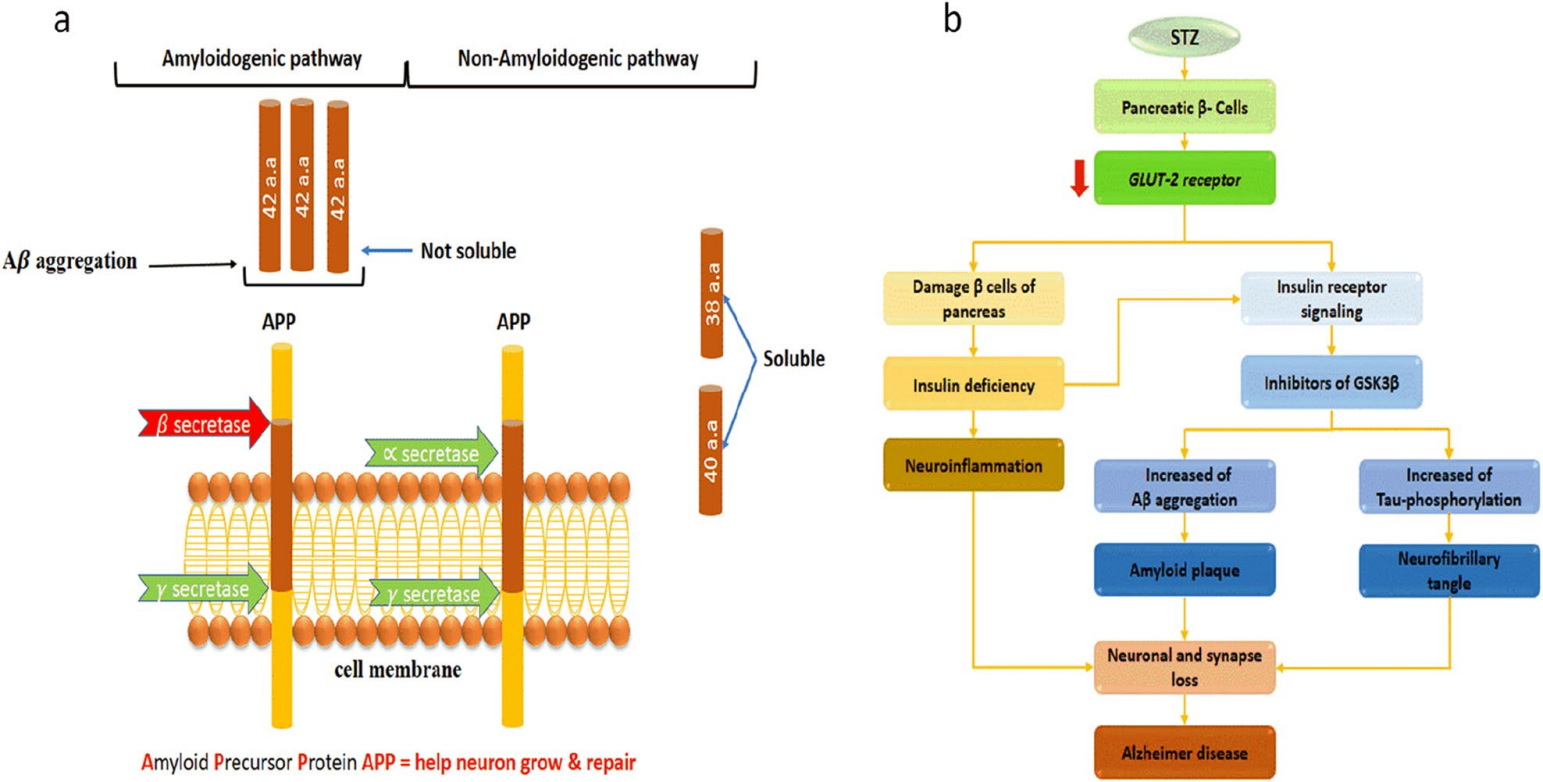
(**a**) Illustrates the aggregation of amyloid beta by β secretes between cells (b) Illustrate how streptozotocin (STZ) damages the beta cells of the pancreas and results in Alzheimer’s disease^9^.

Alzheimer’s disease (AD) is a type of dementia that is common among the elderly. This dementia is a brain disorder that affects the ability to carry out daily activities. This dementia leads to neuron death, and the patient dies between 3 and 9 years. AD affects about 45 million people worldwide and is ranked as the fifth most dangerous disease that leads to death globally^1^. In the United States, about 5.8 million people have Alzheimer's disease, and it is expected to increase in the coming years to 13.8 million by 2050^2^. It is also predicted that 18.9 million people in Europe and 36.5 million people in East Asian countries will suffer from Alzheimer's disease by 2050^1,3^. This pathology was carried out because of the aggregation of amyloid beta (Aβ) and tau protein. Aβ refers to the amino acid sequence length found in the cerebral and peripheral tissue. The sequences of Aβ consist of 36–43 amino acids, and Aβ-42 is the worst one that leads to the aggregation of amyloid plaques^4^. The aggregation levels of Aβ-42 refer to the pathogenesis of Alzheimer’s disease. The second characteristic of AD is the aggregation of tau proteins. This type of protein belongs to the family of soluble proteins and is generated by the gene microtubule-associated protein tau (MAPT). It’s founded on axons of the neuron cells to maintain stability and enhance polymerization. In AD, tau protein aggregation is insoluble and called neurofibrillary tangles, as shown in Fig. 1a^5^.

Insulin in the central nervous system (CNS) controls multiple functions such as eating, learning, behaviors, cognitive functions, etc. Also, it protects the brain from neuroinflammation and redox stress^6,7^. So, neuronal insulin resistance could contribute to appear Alzheimer’s disease^8^. So, streptozotocin is used to induce Alzheimer’s disease experimentally. Streptozotocin or streptozocin (STZ) is a glucosamine–nitrosourea compound that originated as an antibiotic. It is particularly toxic to the pancreas’s insulin, which produces animal beta cells. It is used in experimental research to produce models of animals injured with Alzheimer’s or diabetes type 1 or 2. As shown in Fig. 1b, Injection of STZ intraperitoneal or intracerebroventricular leads to the production of the amyloid-beta (Aβ) fragments and tau protein. Also, produce biochemical alteration, neuroinflammation, and oxidative stress. Aβ aggregation and tau proteins are the main features of the pathological disease, which lead to cognitive function loss and cause neuron death^9^. Zhang et al. report that STZ-induced deficiency in the insulin with AD transgenic mouse model produces β-amyloidogenic in vivo and in vitro models^13^. STZ used in vitro experiments to model cellular processes in Alzheimer’s disease^14,15^. Furthermore, Kamat et al. reported the role of STZ in damaging pancreatic beta cells, which leads to a deficiency of insulin. Also, STZ-impaired insulin in the brain’s receptors causes alteration of glycogen synthase Kinase (GSK3)^9^, affecting the activity of GSK3, which is associated with brain disorders such as Alzheimer’s disease, diabetes type 2, and other brain disorders^10^. High-dose STZ injection strongly affects GLUT-2 of pancreatic beta cells, resulting in loss of insulin secretion^11^. Low doses of STZ, it introduces diabetes type 2^12^.

The neuroplasmonics technique is used to investigate the effect of STZ on the neuron cell’s activities. Neuroplasmonics is a novel optical technique for neural signal recording and imaging that uses SPR sensing to record neuronal activity^16^. This technique depends on the interaction between the electromagnetic fields and free electrons on the gold surface to generate the SPR spectrum, which is linked with the biosensors^17^. This technique provides data for individual neurons in vivo and in vitro with high spatial and temporal resolutions^18^. The benefits of neuroplasmonics are real-time, non-toxic, highly sensitive, offering label-free detection, and providing quantitative and qualitative data on the sensor’s sensitivity^19^.

The Neuroplasmonics technique combining SPR sensing and fluorescence microscopy has been introduced. In this method, EOS supports detecting neural activities in vivo and in vitro by utilizing activity-dependent fluorescence proteins such as calcium indicators and voltage-sensitive fluorescence proteins^20,21^. The reason behind the development of the optical probe is its ability to convert physiological signals into fluorescence changes and appropriate equipment for fluorescence detection. Also, its ability to cooperate with genetic alteration techniques makes it a valuable tool for investigating the neural signal from the tested neurons in living animals and cultures^20,22,23^. In the Neuroplasmonic technique, the dielectric constant of the sensor surface will change when the cell membrane contacts directly with the metal surface. So, the ion redistribution (Na^2+^, K^+^) around the bilayer affects the oscillation activity of the free electrons and generates the SPR signals^24^. SPR sensing is based on detecting the reflective index or changes in the layer thickness^25,26^. Thus, neural activity detection is based on the membrane refractive index and the minimum change in the cellular volume^18,27–34^.

Based on the facts mentioned above, we have introduced a one-dimensional nanograting polycarbonate substrate coated with a thin layer of gold that supports SPR and tunable PIT controlled by an external voltage supply as a new kind of electro-plasmonic sensor. The prepared chips were cultured by Human Nucleus Pulposus Cells (HNPC) purchased from Royan institute. These cells were injected with three different concentrations of STZ (0.5, 1, 2 mM) to induce Alzheimer’s disease by affecting insulin resistance and these concentrations were based on the amount reported by Bagaméry et al.‏^8^. Furthermore, to increase the sensor sensitivity, tunable PITs are used with different polarization^35^. The experiment focused on measuring the effect of STZ on the cultured neuron cell’s activity (inhibition) as a second experiment after this chip successfully measured the activation of neuron cells by using the chemical stimulus (dopamine). The results were recorded using spectroscopy and show good results as a new kind of neuron activity sensing.

## Experimental methods

**Figure 2:**
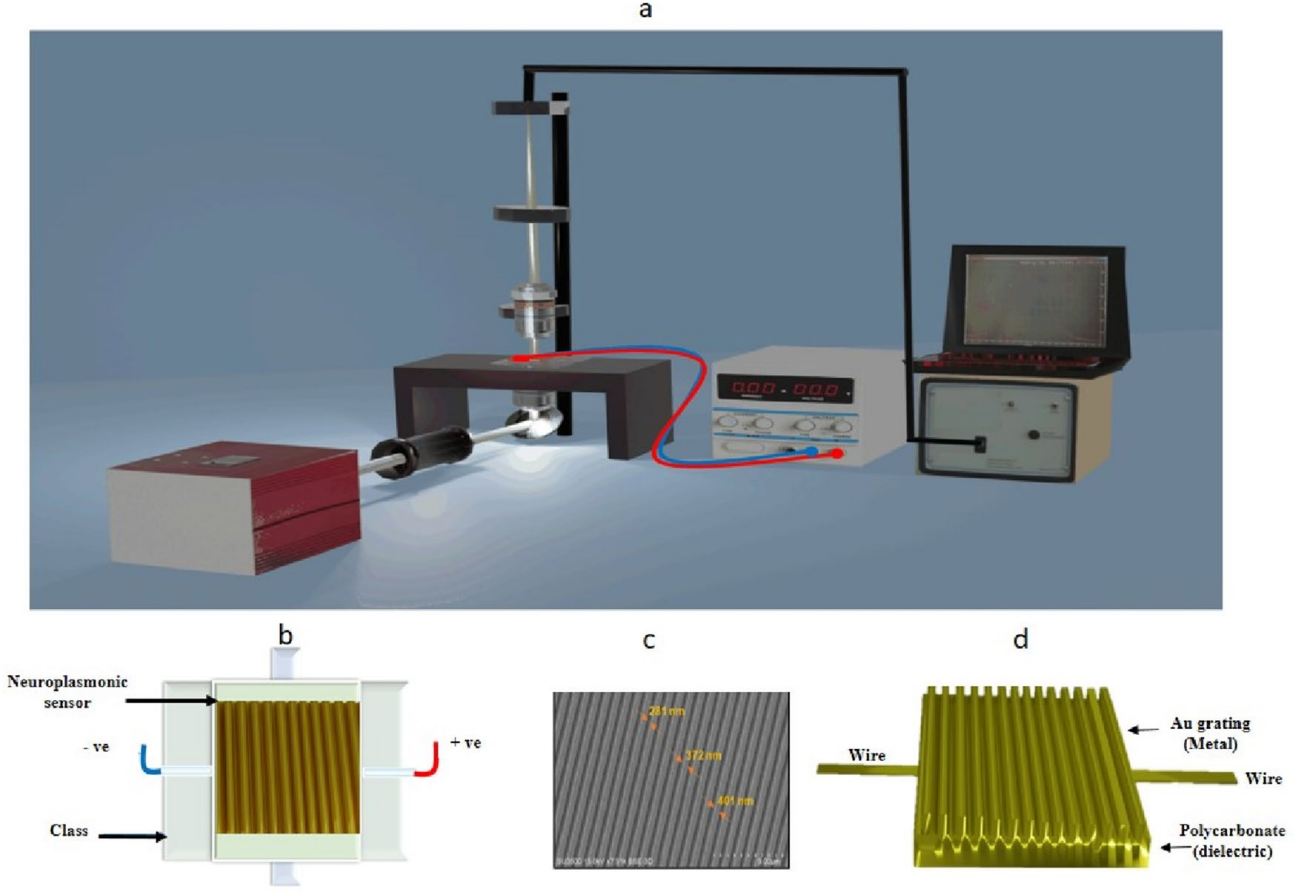
(**a**) The setup of our measurement system which contains the following parts: Light source broadband halogen illuminator (Thorlabs—OSL2), Collimator (Thorlabs—OSL2COL), Polarizer (Thorlabs—GT10-A), Optical lens (Thorlabs—AC254-035-A1), Reflected lens (Mirror), Numerical objective (NA = 0.65), XYZ Translation Stage (Thorlabs—PT3/M), Fiber Adapter Plate with C-Mount (Thorlabs—CMTSMA), Optical fiber (Ocean Optics—QP600-2-VIS–NIR), Spectrometer (Ocean Optics—Nanocalc-XR model), and DC power supply RXN − 303D (**b**) the homemade chamber including the plasmonics chip, (**c**) The scanning electron microscopy (SEM) image of the chip, and (**d**) The schematic of one-dimensional grating nano-gold.

Prepare One-dimensional (1D) plasmonics chips using grating polycarbonate (pc) extracted from a commercial DVD. The first step is using a sharp tool to separate the outer and inner layers. Discarding the outer layer and using the inner layer with a lattice groove. The next step is to remove the purple dye using ethanol, wash it with deionized (DI) water, and then use a desiccator to dry it. Divided the prepared layer (polycarbonate) into main stamp pieces specialized with a one-dimensional pattern. Due care was necessary through the upper steppes to keep the substrate from any contaminants or fingerprints that would negatively affect the measurements. Connect the substrate with two wires and finally coat it with a thin layer of gold using a sputtering machine. The one-dimensional plasmonics chip was prepared now and Fig. 2c shows this chip's scanning electron microscopy (SEM) image. Also, it can be shown schematically in Fig. 2d. This method's advantage is a fast fabrication, cheap, small in size, and biocompatible.

A chamber is required to add the prepared chip into the setup and the food for the cultured cells. So, the homemade chamber is fabricated using classes, as shown in Fig. 2b. After cultured with nerve cells, the chip should be placed inside the home chamber and used to perform the measurement steps. To culture the plasmonics chip with neuron cells, it must be coated with laminin (33 μg/mL, Invitrogen) and then poly-l-lysine to make the cells adhesion^29^. The cultured neuron cells are needed in a glucose medium known as Dulbecco’s modified eagles’ medium (DMEM), which supports the growth of neuron cells.

This medium was supplemented with 20% of Fetal Bovine Serum (FBS), and 100 mg/mL streptomycin at a body temperature (37 °C) in a humidified environment of 5% CO2. The cultured neuron cells of Human Nucleus Pulposus Cells (HNPC) for the first time were grown in small dishes with a medium consisting of neurobasal from Invitrogen, B-27 supplemented (2% w/v), penicillin/streptomycin (100 U/mL) at a body temperature (37 °C) in a humidified environment of 5% CO_2_, and l-glutamine (500 μM). When the neuron cells are cultured successfully in the plasmonics chip, streptozotocin (STZ) is added in three different concentrations by dividing the samples into four groups. The first group remains without adding STZ. The second group added a low concentration of about 0.5 mM of STZ to the cultured cells. The third group added a middle concentration of about 1 mM of STZ to the cultured neuron cells. Finally, adding a high concentration of about 2 mM of STZ to the remaining group.

Adding STZ and treatment of the cells by that for 10 days must reduce the activity of the cells, as mentioned previously. The neural activity detection is based on the membrane refractive index and the minimum change in the cellular volume, which shifts the plasmonic sensor's resonance. Culturing the neuron cells on the gold surface changes the refractive index because the cell membrane alters the dielectric constant of the sensor surface. The sensing is based on detecting the reflective index or changes in the layer thickness.

The chip characterization is important before being cultured with neuron cells. The chip was excited, as shown in Fig. 2a. Using a halogen source (broadband) with a wavelength ranging from 400 nm to 700 n and passing through a polarizer. An objective lens will focus on the polarized light with NA = 0.65. On the interface between the dielectric (grading polycarbonate) and metal (grading gold film with a thickness of 35 nm), the SPPs were measured by spectroscopy. With SPPs, an amazing physical phenomenon appears as a sharp transparency window inside a vast absorption spectrum.

This phenomenon is known as plasmonic induce transparency (PIT), which originates from the strong destructive interference between wideband states (super radiant mode) and narrowband states (sub-radiant mode) in plasmonic nanostructures. These characteristics are useful for employing optical sensors and enhancing sensor sensitivity. Also, applying an external voltage to the chip will increase the plasmonics spectrum's peaks and the chip's sensitivity^35^.

To stimulate the cultured neurons and record the activity of the cells, the cultured chip is placed inside the housing chamber and injected with cell food (DMEM) to maintain the normal activity of the cells.

In the first step, measure the activity of the cells in the first groups (without STZ) in both P and S polarization and apply external voltage (1, 2, 2.5, 3 V) to each case. In the next steps, measure the activity of the cells by repeating the upper steps with the other groups with different concentrations of STZ (low, middle, high).

### Statements

The rat neural cells were purchased from Avicenna Research institute.

Human Nucleus Pulposus Cells were purchased from Royan Institute (https://www.royan.org/en/).

## Results and discussion

**Figure 3:**
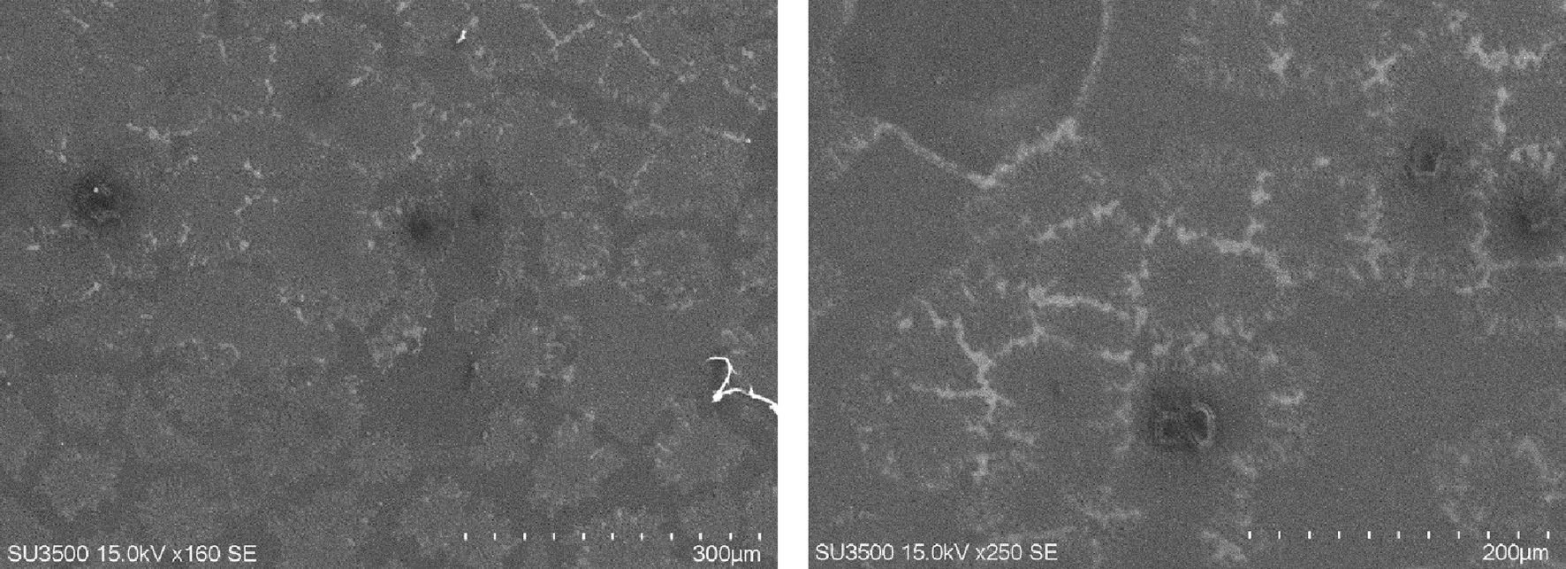
The microscopic image shows the neuron cells were successfully cultured on the plasmonic grating chip.

The tunable electro-plasmonic chip was cultured with neuron cells and investigated the ability of STZ to induce AD. The neuron cells are successfully cultured on the electro-plasmonic chip and injected with STZ in different concentrations. The scanning electron microscopy (SEM) image of the cultured cells is illustrated in Fig. 3.

The experiment steps included samples without and with STZ in different concentrations (0.5, 1, 2 mM). Also, the measurement procedures included applying different voltage levels (1, 2, 2.5, and 3) V on the chip and using S and P polarizations.

**Figure 4:**
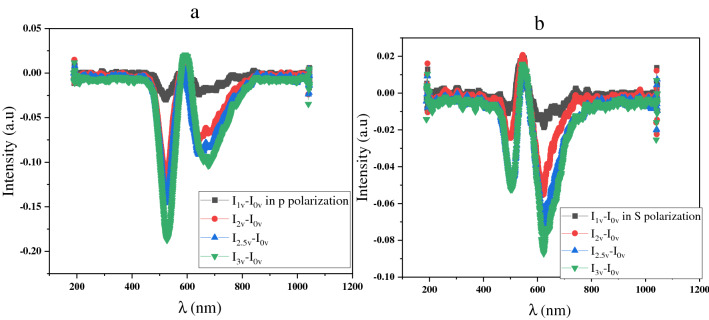
Show the measurement of the activity of the cells without STZ (**a**) with P polarization (**b**) with S polarization.

The first step is measuring the neuron cell’s activities without STZ and applying external voltage with P and S polarizations. The results are shown in Fig. 4. The results show that we have successfully investigated the tunable electro-plasmonic sensor. The sensor's sensitivity has been increased by increasing the applied voltage from 1 to 3 V. The plasmonic mode of the spectrum intensity in P polarization has a clear shifting at 520 nm and 620 nm with increasing applied voltage.

Also, it shows that the sensing performance with P polarization is better than S polarization. The results of S polarization Fig. 4b show the same spectrum at 520 nm with different applied voltages. The other measurements also show that the spectrum intensity clearly contrasts several modes in P and overlaps with S polarization. The electro-plasmonics sensor shows a good performance for sensing the cell’s activity without affecting STZ.

It is widely known that stern layer voltage is close to the plasmonic chip and the neural tissue. Therefore, there is stern layer voltage, and thus electrical double layer consists of Thomas–Fermi screening length in the metallic layer and stern layer thickness in the electrolyte. These double layers are sensitive to variations in the potassium or sodium levels in the neural area, which results in plasmonic mode alterations^30^.

As mentioned early in Fig. 1b, STZ is toxic to the pancreatic beta cells and usually GLUT-2. So, STZ induced dysregulation of insulin and glucose in the cultured cells model^8^. The GLUT-2 is the main glucose transporter in pancreatic beta cells and plays an important role in insulin secretion from beta cells^36^. The insulin receptor (IR) and the Insulin-like growth factor-1 receptor (IGF-1R) signaling (IIR) are markedly disturbed in the central nervous system (CNS) of Alzheimer’s disease patients^37,38^. Furthermore, injection of the STZ will decrease glucose expression of GLUT-2^39^, reduce the expression of insulin receptors^40^, decrease the levels of phosphorylated GSK-3^14^, and induces insulin resistance^41,42^. Increased apoptosis^43,44^. Insulin resistance occurs when the cell’s tissues fail to respond to the insulin. Moreover, binding the STZ to the insulin receptor will decrease the neuron’s activity. The neuron activity is done by doping Na^+^ channels or opening K^+^ channels. Since the electrical double layer is primarily affected by these variations in K^+^ and Na^+^ in neurons, it serves as the primary modulation source for neurons and neural activity. These activities will reduce because of the effect of STZ, as mentioned above.

**Figure 5:**
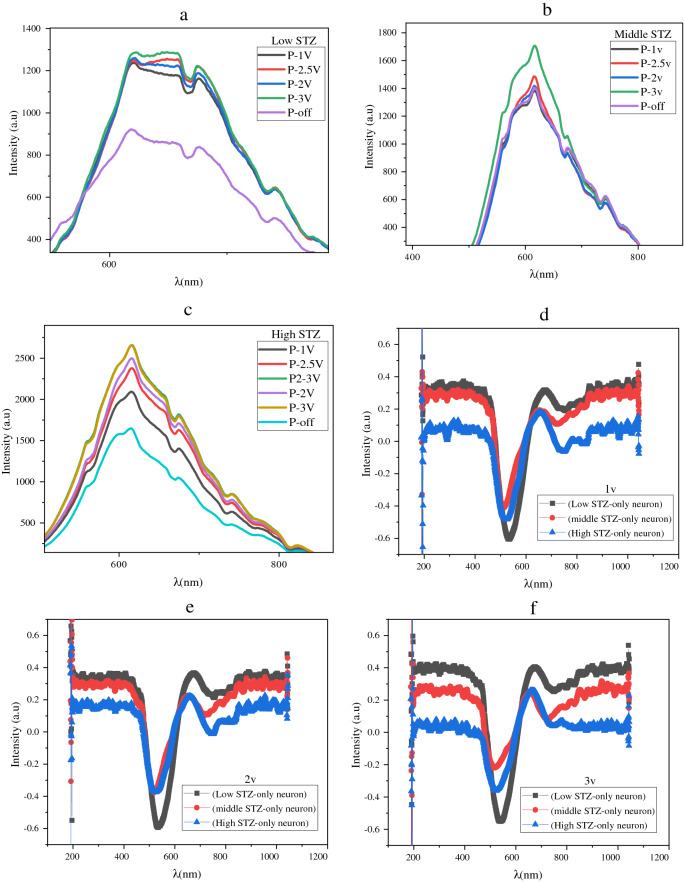
Show the activity of the cells under P polarization by applying an external voltage (**a**) With a low concentration of STZ, (**b**) With a middle concentration of STZ, (**c**) With a high concentration of STZ, (**d**) with applying 1 V and different concentration of STZ, (**e**) with applying 2 V and different concentration of STZ, (**f**) with applying 3 V and different concentration of STZ.

Figure 5 shows the spectrum measurement of the neuron activity under the different concentrations of STZ. The results refer to the response of the cultured neuron cell’s activity to the effecting of STZ. Figure 5a–c shows that increasing the applied voltage from 0 to 3 V in P polarization will increase the sensor sensitivity. Also, these measurements show the high sensitivity of the electro-plasmonics sensor to the neuron cell’s activities, and the performance enhances by increasing the applied voltage to 3 V.

The second measurement is shown in Fig. 5d–f which appear the effect of different concentrations of STZ on the cell’s activity. Furthermore, increasing the concentration of the STZ to the middle (1 mM) and then to a high concentration (2 mM). The results show high accuracy in measuring the activity of the cells under the effecting of STZ in P polarization and enhancing the performance of sensing by increasing the applied voltage. Also, the results show the electro-plasmonics sensor's ability to sense the cells' activity under different concentrations of STZ by applying external voltage from 1 V, 2 V, and 3 V, respectively. The results show that the spectrum intensity reduces with increasing the concentration of STZ at the wavelength 520 nm and 620 nm. The results refer to the electro-plasmonics sensor's good performance for sensing the stimulation and inhabitation (under the effecting of STZ) of the neuron cells.

In other words, adding the STZ to the cultured neuron cells led to a change in the distribution of ions, as explained before as double-layer formation and change. As a result, the plasmonic interface was affected, and the activity of the cells decreased with increasing the STZ concentration to 2 mM.

**Figure 6:**
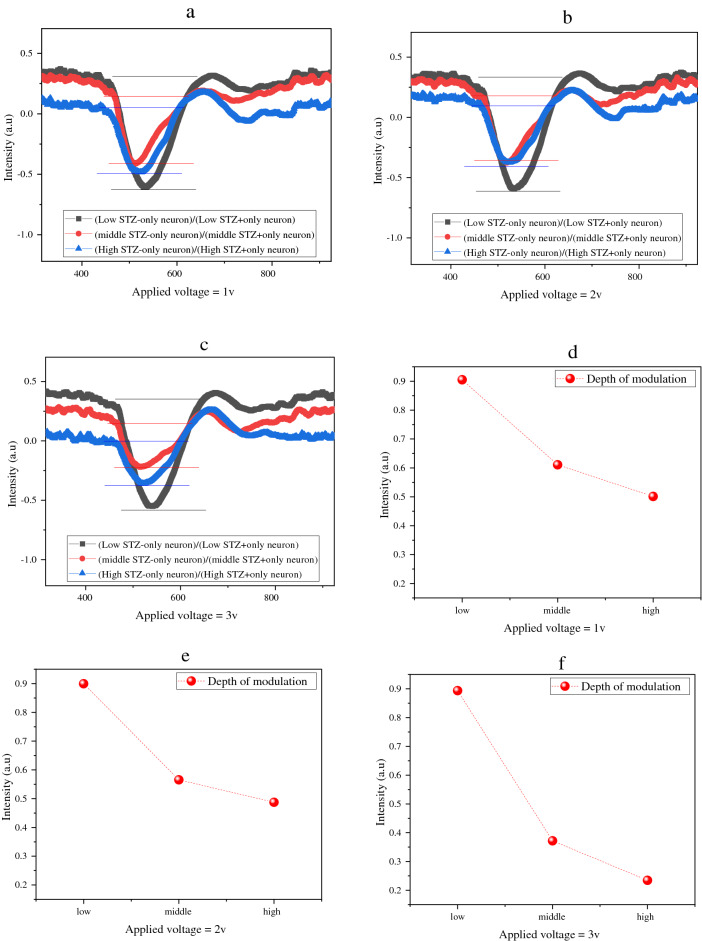
Sensing diagram of the samples under low, middle, and high concentrations of STZ in 620 nm as one of the main modes.

This electro-plasmonic sensor can sense the different effecting concentrations of STZ on the activity of the cells in the transient process of an electrical signal between one neuron to the next by chemical action. The sensing diagram of the sensor can plot in all of the main modes, such as 620 nm, as shown in Fig. 6. The modulation depth shows the clear difference between the different concentrations.

The spectrum amplitude depends on changes in the refractive index of the cultured neuron cells. The change in the refractive index occurs from the activity of the cultured neuron cells. In the extracellular space, sodium (Na^+^) and chloride (Cl^−^) ions are more concentrated, and inside the cells, the potassium (K^+^) and organic anions (proteins and amino acids) exist. The sodium–potassium pump family actively couples the efflux of three ions of Na^+^ with the influx of two ions of K^+^ depending on an ATP manner. At the same time, the chloride symporter by gradient k^+^ could be a couple of the efflux Cl^−^ and K^+^ against the gradient concentration of Cl^-^ without using ATP. These gradients concentration with protein activities produce the steady state represented by negatively charged intracellular compared to the extracellular. Because of the cellular membranes' selective permeability to ions and the negatively charged intracellular environment, an increase in extracellular K^+^ changes the ion gradient, reversing K^+^ outflow and boosting permeability to Na^+^ (voltage-gated channels). This results in membrane depolarization, frequently utilized to research electrical activity-dependent alterations in neuron cells.

It is obvious from the molecular assays of the cells with STZ incorporation that the STZ produces more similar characteristics pathology of sporadic AD. This fact comes from the single transfiguration of genes related to sporadic AD. In addition, neuroinflammation of the cells has been implicated as a leading risk factor which is one of the main reasons for sporadic AD. Based on this fact, STZ can produce similar properties of sporadic AD which can be used to discover the underlying pathophysiological mechanism.

**Figure 7:**
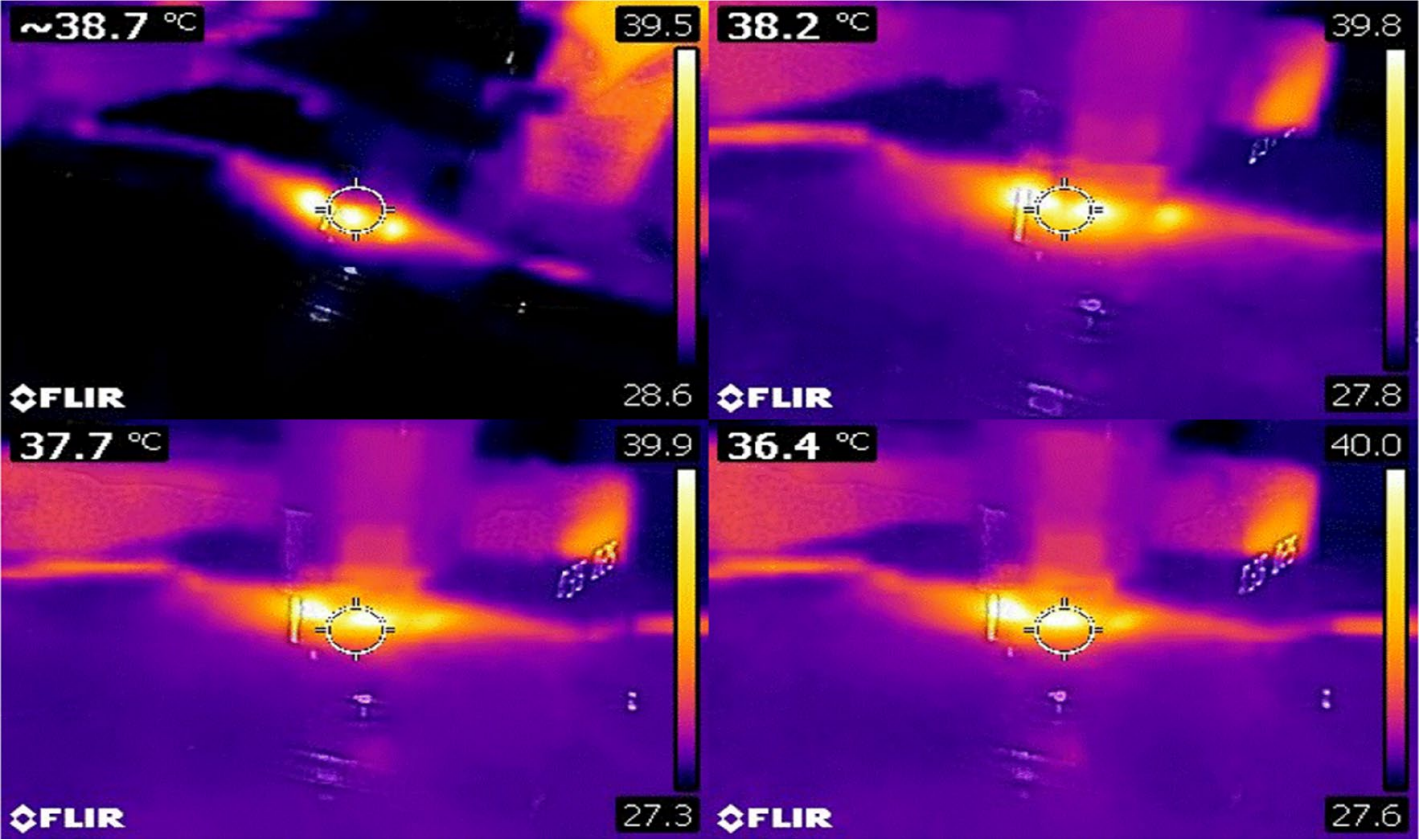
Illustrate the temperature of the cultured cells under the effect of plasmonic taken by an IR camera.

One of the main challenges in dealing with neuron cells is the temperature. Because increasing the temperature will leads to damage to the cultured neuron cells. So, for this reason, an IR camera is used to measure the temperature of the cultured neuron cells, which shows normal temperature and is safe to keep the cell’s activities, as shown in Fig. 7.

## Conclusion

This paper investigates the ability of the electro-plasmonics chip to sense the effect of the STZ on neuron cell activity using a one-dimensional gold nano grating chip by applying an external voltage with P polarization. The results show a high ability of the electro-plasmonic chip to sense the activity of the cultured neuron cells. The sensitivity performance will increase by increasing the externally applied voltage to 3 V. In addition, it has a high ability to measure the degradation in neuronal activity caused by the addition of STZ. The change in the measured spectrum comes from the cell’s depolarization as well as changes in the ion distribution of the cultured cells that would affect the plasmons interface. When the plasmon interface changes, the refractive index changes because the contrast of the cells changes. The results indicate the high sensitivity of the electro-plasmonic one-dimensional nanograting gold sensor to sense the activity of cultured neuron cells with different concentrations of STZ. Moreover, the suggested electro-plasmonic chip is a promising sensor for future cultured neuron cell experiments. It could be used in a biomedical research laboratory to measure the effectiveness of suggested drugs on the neuron cell’s activity.

## Data Availability

Data underlying the results presented in this paper can obtain from the corresponding author (m_hamidi@sbu.ac.ir) upon request.

## References

[1] Prince MJ, Wimo A, Guerchet MM, Ali GC, Wu Y-T, Prina M (2015). World Alzheimer Report 2015: The Global Impact of Dementia: An Analysis of Prevalence, Incidence, Cost and Trends.

[2] Alzheimer’s disease facts and figures. *Alzheimers Dement.* 391–460 (2020).‏

[3] Canevelli M, Lacorte E, Cova I, Zaccaria V, Valletta M, Raganato R (2019). Estimating dementia cases amongst migrants living in Europe. Eur. J. Neurol..

[4] Masters CL, Selkoe DJ (2012). Biochemistry of amyloid β-protein and amyloid deposits in Alzheimer disease. Cold Spring Harb. Perspect. Med..

[5] Amini M (2022). Plasmonics optoelectronics nanobiosensors for detection of alzheimer’s disease biomarker via amyloid-beta (Aβ) in near-infrared. Plasmonics.

[6] Kullmann S (2016). Brain insulin resistance at the crossroads of metabolic and cognitive disorders in humans. Physiol. Rev..

[7] Santiago JCP, Hallschmid M (2019). Outcomes and clinical implications of intranasal insulin administration to the central nervous system. Exp. Neurol..

[8] Bagaméry F (2020). Lack of insulin resistance in response to streptozotocin treatment in neuronal SH-SY5Y cell line. J. Neural Transm..

[9] Kamat PK (2015). Streptozotocin induced Alzheimer's disease like changes and the underlying neural degeneration and regeneration mechanism. Neural Regen. Res..

[10] Salkovic-Petrisic M, Hoyer S (2007). Central insulin resistance as a trigger for sporadic Alzheimer-like pathology: an experimental approach. Neuropsychiatr. Disord. Integr. Approach.

[11] Wu J, Yan L-J (2015). Streptozotocin-induced type 1 diabetes in rodents as a model for studying mitochondrial mechanisms of diabetic β cell glucotoxicity. Diabetes Metab. Syndrome Obes. Targets Ther..

[12] Wang Z, Gleichmann H (1998). GLUT2 in pancreatic islets: Crucial target molecule in diabetes induced with multiple low doses of streptozotocin in mice. Diabetes.

[13] Zhang Y (2015). Geniposide attenuates insulin-deficiency-induced acceleration of β-amyloidosis in an APP/PS1 transgenic model of Alzheimer’s disease. Neurochem. Int..

[14] Plaschke K, Kopitz J (2015). In vitro streptozotocin model for modeling Alzheimer-like changes: Effect on amyloid precursor protein secretases and glycogen synthase kinase-3. J. Neural Transm..

[15] Guo X (2016). Small molecule LX2343 ameliorates cognitive deficits in AD model mice by targeting both amyloid β production and clearance. Acta Pharmacol. Sin..

[16] Sohrabi F, Hamidi SM (2016). Neuroplasmonics: From Kretschmann configuration to plasmonic crystals. Eur. Phys. J. Plus.

[17] Benounis M (2015). High sensitive surface plasmon resonance (SPR) sensor based on modified calix (4) arene self-assembled monolayer for cadmium ions detection. Mater. Trans..

[18] Zhang J, Atay T, Nurmikko AV (2009). Optical detection of brain cell activity using plasmonic gold nanoparticles. Nano Lett..

[19] Sohrabi F (2020). Membrane activity detection in cultured cells using phase-sensitive plasmonics. Opt. Express.

[20] Kim SA, Jun SB (2013). In-vivo optical measurement of neural activity in the brain. Exp. Neurobiol..

[21] Grienberger C, Konnerth A (2012). Imaging calcium in neurons. Neuron.

[22] Mutoh H (2011). Optogenetic monitoring of membrane potentials. Exp. Physiol..

[23] Cao G (2013). Genetically targeted optical electrophysiology in intact neural circuits. Cell.

[24] Choi SH, Kim SJ, Im C-H, Kim SA, Kim D (2011). Quantitative model for the change of optical resonance in neural activity detection systems based on surface plasmon resonance. Opt. Laser Technol..

[25] Knoll W (1998). Interfaces and thin films as seen by bound electromagnetic waves. Annu. Rev. Phys. Chem..

[26] Raether H (1988). Surface Plasmons on Gratings. Surface Plasmons on Smooth and Rough Surfaces and on Gratings.

[27] Huang Y (2013). Theoretical analysis of voltage-dependent fiber optic surface plasmon resonance sensor. Opt. Commun..

[28] Kim SA (2012). In vivo optical neural recording using fiber-based surface plasmon resonance. Opt. Lett..

[29] Kim SA (2008). Optical measurement of neural activity using surface plasmon resonance. Opt. Lett..

[30] Choi SH (2011). Quantitative model for the change of optical resonance in neural activity detection systems based on surface plasmon resonance. Opt. Laser Technol..

[31] Stepnoski RA (1991). Noninvasive detection of changes in membrane potential in cultured neurons by light scattering. Proc. Natl. Acad. Sci..

[32] Cohen LB, Keynes RD, Hille B (1968). Light scattering and birefringence changes during nerve activity. Nature.

[33] Foust AJ, Rector DM (2007). Optically teasing apart neural swelling and depolarization. Neuroscience.

[34] Teramura Y, Arima Y, Iwata H (2006). Surface plasmon resonance-based highly sensitive immunosensing for brain natriuretic peptide using nanobeads for signal amplification. Anal. Biochem..

[35] Kadhim HJ (2022). Tunable plasmon induced transparency in one-dimensional gold nano-grating as a new kind of neuro-transmitter sensor. Optik.

[36] Wu L, Fritz JD, Powers AC (1998). Different functional domains of GLUT2 glucose transporter are required for glucose affinity and substrate specificity. Endocrinology.

[37] Frölich L (1998). Brain insulin and insulin receptors in aging and sporadic Alzheimer's disease. J. Neural Transm..

[38] Moloney AM (2010). Defects in IGF-1 receptor, insulin receptor and IRS-1/2 in Alzheimer's disease indicate possible resistance to IGF-1 and insulin signalling. Neurobiol. Aging.

[39] Biswas J (2016). Streptozotocin induced neurotoxicity involves Alzheimer’s related pathological markers: A study on N2A cells. Mol. Neurobiol..

[40] Wang P (2011). The n-terminal 5-MER peptide analogue P165 of amyloid precursor protein exerts protective effects on SH-SY5Y cells and rat hippocampus neuronal synapses. Neuroscience.

[41] Steen E (2005). Impaired insulin and insulin-like growth factor expression and signaling mechanisms in Alzheimer's disease: Is this type 3 diabetes?. J. Alzheimer's Dis..

[42] Agrawal R (2011). Insulin receptor signaling in rat hippocampus: A study in STZ (ICV) induced memory deficit model. Eur. Neuropsychopharmacol..

[43] Rajasekar N (2014). Protection of streptozotocin induced insulin receptor dysfunction, neuroinflammation and amyloidogenesis in astrocytes by insulin. Neuropharmacol..

[44] Biswas J (2017). Streptozotocin alters glucose transport, connexin expression and endoplasmic reticulum functions in neurons and astrocytes. Neuroscience.

